# Supplementary figures and images for: Biology and engineering of integrative and conjugative elements: Construction and analyses of hybrid ICEs reveal element functions that affect species-specific efficiencies
Source: PLoS Genet. 2022 May 18;18(5):e1009998. doi: 10.1371/journal.pgen.1009998 (PMC9154091; doi:10.1371/journal.pgen.1009998)

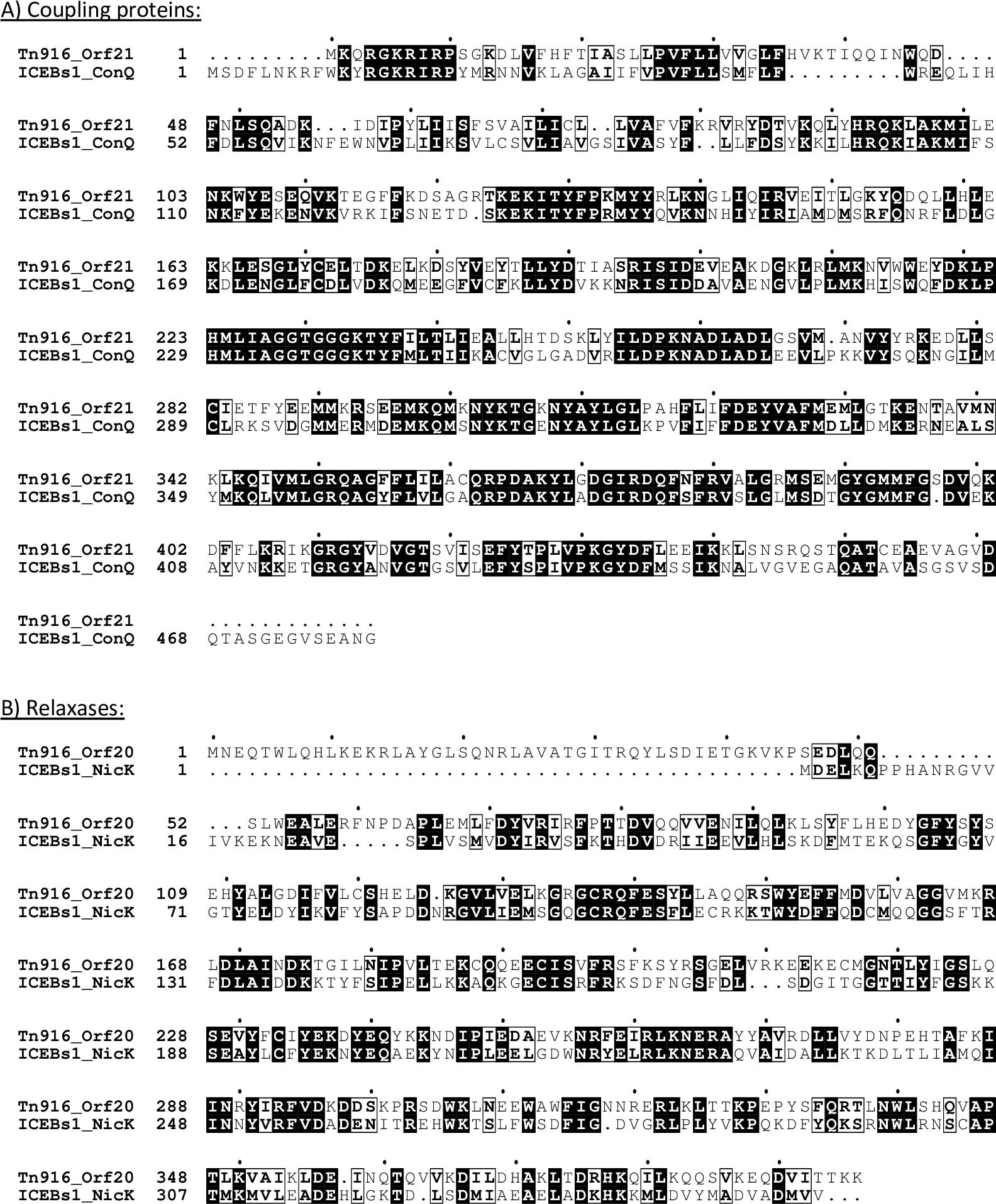

Supplement: S1 Fig — Protein sequences were aligned with the Needleman-Wunsch algorithm (https://www.ebi.ac.uk/Tools/psa/emboss_needle/) [94]. The output alignment was shaded with ESPript 3.0 (https://espript.ibcp.fr/ESPript/cgi-bin/ESPript.cgi) [95]. Black shading indicates identical residues; boxes indicate similar residues. A similar alignment of these relaxases has previously been reported [14]. (TIF) [file pgen.1009998.s001.tif]
