# Supplementary material for: Biology and engineering of integrative and conjugative elements: Construction and analyses of hybrid ICEs reveal element functions that affect species-specific efficiencies
Source: PLoS Genet. 2022 May 18;18(5):e1009998. doi: 10.1371/journal.pgen.1009998 (PMC9154091; doi:10.1371/journal.pgen.1009998)
Supplement: S1 Table — This table includes information about the location and stability of different ICEs in transconjugants after transfer from specific donors. Elements and respective donors include: Tn916 (ELC1566), ICEBs1-tetM (ELC1795), H1-tetM (ELC1722), and H2-tetM (ELC1725). (PDF) [file pgen.1009998.s002.pdf]

S1 Table. Mapping ICE integration sites in transconjugants.

Recipient species[a]	TCs	ICE transferred [b]			
		Tn*916*	ICE*Bs1-tetM*	H1-*tetM*	H2-*tetM*
B. subtilis	Stable	100%	100%	100%	100%
	#1	GTTTTTTAACTTAAAAAAATAT (nupQ_maeN)	CTAGGTTGAGGGCCTAG (*trnS-leu2*)	CTAGGTTGAGGGCCTAG (*trnS-leu2*)	CTAGGTTGAGGGCCTAG (*trnS-leu2*)
	#2	TTATTGCTTATTTTATTGGAAA (desK)	CTAGGTTGAGGGCCTAG (*trnS-leu2*)	CTAGGTTGAGGGCCTAG (*trnS-leu2*)	CTAGGTTGAGGGCCTAG (*trnS-leu2*)
	#3	TTTTTATGTATAAAAAAAGACA (yqeY_yqeZ)	CTAGGTTGAGGGCCTAG (*trnS-leu2*)	CTAGGTTGAGGGCCTAG (*trnS-leu2*)	CTAGGTTGAGGGCCTAG (*trnS-leu2*)
E. faecalis	Stable	100%	5%	4%	3%
	#1	GTAAAAGGGCGTTTTTTTATAA (intergenic)	Circular	Circular	Circular
	#2	ATTTTAAAGATAAAAAATTCTT (HK)*	Circular	Circular	Circular
	#3	TTCCATTAATTTTTAATATCAA (intergenic)	Circular	Circular	Circular
E. caccae	Stable	100%	ND < 1%	3%	2%
	#1	AAATTTTGATATTTTTTATACA (intergenic)	Circular	Circular	CAAGTATTATGGTCCTG (*pepF*)
	#2	CAAATACGGCAACTTTTTTTTA (intergenic)	Circular	Circular	GCTAGGTGTTGGACCAG (ADH)**
	#3	ACAGATCAATGACAATTTAATT (intergenic)	Circular	Circular	Circular
E. durans	Stable	100%	3%	5%	8%
	#1	ATTTTTTGTGTAAAAAAAAATAC (intergenic)	Circular	Circular	ACTAGTCCAAGGACTTG (DAHP)
	#2	TAAATACTTATCCTGATGGAAA (HP)	Circular	Circular	CTAGGAGCGGGACTTTT (AAP)
	#3	GAACGATATGTTTTTTTTATAT (intergenic)	Circular	Circular	TCCGGTCGCAGGACTAG (SDH)

[a]Donor strains contained these elements: Tn*916* (ELC1566), ICE*Bs1-tetM* (ELC1795), H1-*tetM* (ELC1722), and H2-*tetM* (ELC1725). All donors were D-alanine auxotrophs (*alr*::*cat*) for counter-selection of transconjugants during mating assays. ICE*Bs1*, H1, and H2

donors also contained *amyE*::[(Pspank(hy)-*rapI*) *spc*] for IPTG-inducible overproduction of RapI to stimulate element gene expression and subsequent excision.

[b]The genetic stability of these elements in transconjugants was evaluated. One hundred transconjugants were re-streaked non-selectively and then patched to check for tetracycline resistance (indicative of ICE presence). Arbitrary PCR was used to map ICE insertion sites in three isolates that had maintained their elements from each mating pair (TC #1-3 sites). “Circular” indicates that circular ICEs were detected by PCR rather than a chromosomal insertion site. For the detected integration sites, a genomic context of 26 bp (Tn*916* insertions) or 17 bp (ICE*Bs1*, H1, H2 insertions) is shown. The genes or predicted gene products of insertion sites are indicated, unless the site is in a predicted intergenic region. Gene key: HK = histidine kinase, DAHP = DAHP synthase, AAP = amino acid permease, SDH = serine dehydratase, HP = hypothetical protein. *indicates last 10% of ORF. **indicates this site was also identified previously [1].

1. Brophy JAN, Triassi AJ, Adams BL, Renberg RL, Stratis-Cullum DN, Grossman AD, et al. Engineered integrative and conjugative elements for efficient and inducible DNA transfer to undomesticated bacteria. Nat Microbiol. 2018 Sep;3(9):1043–53.
